# Host Specificity in the Honeybee Parasitic Mite, *Varroa spp*. in *Apis mellifera* and *Apis cerana*

**DOI:** 10.1371/journal.pone.0135103

**Published:** 2015-08-06

**Authors:** Alexis L. Beaurepaire, Tuan A. Truong, Alejandro C. Fajardo, Tam Q. Dinh, Cleofas Cervancia, Robin F. A. Moritz

**Affiliations:** 1 Institut für Biologie, Martin Luther Universität Halle-Wittenberg, Halle (Saale), Germany; 2 Bee Research and Development Centre, Dong Da Hanoi, Vietnam; 3 UPLB Bee Program, Institute of Biological Sciences, University of the Philippines Los Baños, Los Baños, Philippines; 4 Department of Zoology and Entomology, University of Pretoria, Pretoria, South Africa; University of North Carolina, Greensboro, UNITED STATES

## Abstract

The ectoparasitic mite *Varroa destructor* is a major global threat to the Western honeybee *Apis mellifera*. This mite was originally a parasite of *A*. *cerana* in Asia but managed to spill over into colonies of *A*. *mellifera* which had been introduced to this continent for honey production. To date, only two almost clonal types of *V*. *destructor* from Korea and Japan have been detected in *A*. *mellifera* colonies. However, since both *A*. *mellifera* and *A*. *cerana* colonies are kept in close proximity throughout Asia, not only new spill overs but also spill backs of highly virulent types may be possible, with unpredictable consequences for both honeybee species. We studied the dispersal and hybridisation potential of *Varroa* from sympatric colonies of the two hosts in Northern Vietnam and the Philippines using mitochondrial and microsatellite DNA markers. We found a very distinct mtDNA haplotype equally invading both *A*. *mellifera* and *A*. *cerana* in the Philippines. In contrast, we observed a complete reproductive isolation of various Vietnamese *Varroa* populations in *A*. *mellifera* and *A*. *cerana* colonies even if kept in the same apiaries. In light of this variance in host specificity, the adaptation of the mite to its hosts seems to have generated much more genetic diversity than previously recognised and the *Varroa* species complex may include substantial cryptic speciation.

## Introduction

The Western honeybee *Apis mellifera*, originally native to Europe, Africa, and the Middle East, has been repeatedly introduced in almost all regions of the world due to its importance for apiculture [1]. Introductions into Eastern Asia have been ongoing for over a century with most drastic negative consequences for global beekeeping [2]. Following its introduction, *A*. *mellifera* came into contact with a broad range of parasites and pathogens infecting native Asian honeybees. Among these, *Varroa destructor*, *an ectoparasitic* mite originally a non-lethal parasite of *A*. *cerana*, managed to switch to this introduced non-adapted host [3].

The first observations of a mite spill over from *A*. *cerana* to *A*. *mellifera* were made in Japan in 1957, about 80 years after the introduction of the Western honeybee [4]. A second event of host switch is thought to have taken place in the far east of the former Soviet Union, where they became infested with *Varroa* from Korea [5]. After these successful spill-overs, the mite spread from Japan to the other side of the Pacific Ocean, first in Paraguay and later in Brazil and North America [6]. In parallel, the Korean mite expanded to the West into Europe and today to all other regions of the world suitable for beekeeping with the exception of Australia [3].

At the time these hosts switches were first reported, the genus *Varroa* comprised only three species: *V*. *jacobsoni*, which was believed to be the one which managed to invade *A*. *mellifera colonies*, but also *V*. *rindereri* and *V*. *underwoodi*. However, Anderson and Trueman performed the first large scale survey of the genetic diversity of the mite in Asia using mitochondrial DNA analyses [7] identified a species morphologically and genetically distinct from *V*. *jacobsoni* that they named *V*. *destructor*. This species was originally restricted to the north of Asia, unlike *V*. *jacobsoni* which infects *A*. *cerana* and *A*. *mellifera* in the southern part of the continent [8].

*V*. *destructor* causes little damage at the colony level on its original host, *A*. *cerana*, as it only reproduces in drone brood. Therefore, the mite population can only expand during periods when drones are reared. The damage is limited since the parasites do not interfere with the workers’ pupal development. Furthermore, the grooming performed by the worker bees helps to control the mite populations efficiently. In contrast, the mite successfully reproduces in both drones and worker brood of *A*. *mellifera*. Unfortunately, the Western honeybee has only limited natural defence mechanisms and *V*. *destructor* can kill colonies within few years. In addition to directly harming the host by sucking haemolymph, it also acts as a vector for numerous viruses and spreads these pathogens between individuals and colonies [9]. As a result, *V*. *destructor* is today considered to be the major cause for colony losses of the Western honeybee [10–12].

It is sad to see that the problem may be very sustainable because of the ongoing sympatric management of *A*. *mellifera* and *A*. *cerana* for honey production in Asia. Since both honeybee species are kept next to each other, often in the same apiary, the mite is in close and constant contact with both host species. This not only sets the stage for a continuous spill over potential of mites from *A*. *cerana* to *A*. *mellifera*, but also for a spill back of highly virulent non-native mite types to new *A*. *cerana* hosts which were previously not exposed to the highly virulent *Varroa destructor* lineage on *A*. *mellifera*. This is of concern because *Varroa* mites in *A*. *cerana* show much more variability than those found in *A*. *mellifera*, which have spread as very distinct and almost clonal lineages across the globe [13–14]. Hence, there are a multitude of opportunities to repeat the spill-overs with even more virulent *Varroa* strains perpetuating the problem for global apiculture, not just for *A*. *mellifera* but also for *A*. *cerana*. Therefore, we studied the dispersion routes as well as the spill-over and spill-back potential of *V*. *destructor* between its two hosts in two different regions of Asia using both mitochondrial and microsatellite DNA markers.

## Material and Methods

### Sampling and location

The permits for fieldwork and the local transport for wildlife export were obtained from the Department of Environment and Natural Resources of the Philippines and the Department of Animal Health of Vietnam. The sampling did not involve endangered or protected species.

*V*. *destructor* adult females were sampled directly from capped pupae cells of drones in *A*. *cerana* colonies and from workers and drones pupae cells in *A*. *mellifera* colonies. Mite reproduction was not observed in the worker brood of *A*. *cerana* during the sampling. In *A*. *mellifera*, we found reproducing mites in both drones and workers cells. The sampling was conducted in the apiary of the University of Los Banos (Philippines) and in Dien Bien and Son La (Vietnam) in 2013. In these locations, both honeybee host species were kept in sympatry on the same or on adjacent apiaries, within honeybee flight distance range (<1000m). Additionally, mites were collected in 2013 on the island of Cat Ba (Vietnam) a natural reserve where only *A*. *cerana* occurred. Finally, dead mites were sampled from boards placed at the bottom of three Western honeybee colonies in Lipa city, Philippines in 2015. All mites were directly transferred into 99% ethanol and stored at -20°C shortly after sampling.

The DNA of individual mites was extracted using a standard Phenol-Chloroform protocol [15]. The quality and amount of each DNA extract was determined using a Nanodrop spectrophotometer (Thermo Fisher Scientific Inc., Wilmington, USA). All in all, 372 mites were analysed (263 from 20 *A*. *mellifera* colonies and 109 from 14 *A*. *cerana* colonies) (Table 1).

**Table 1:** Information on the genotyped individuals.

Sampling location	Host	Clusters	*N*_*A*_	*N*_*C*_	*N*_*I*_	N_al_	Allelic richness	H_O_
P-Los Banos	*A*.*c*	Philippines	1	3	25	2.83 ±0.70	2.17 ±0.33	0.17 ±0.07
P-Los Banos	*A*.*m*	Philippines	1	3	6	3.00 ±0.63	2.88 ±0.57	0.10 ±0.07
P- Lipa City	*A*.*m*	Korea	1	3	91	2.50 ±1.02	1.33 ±0.30	0.03 ±0.02
V-Dien Bien	*A*.*m*	Korea	3	8	137	3.00 ±1.44	1.83 ±0.64	0.03 ±0.02
V-Son La	*A*.*m*	Korea	2	6	29	2.00 ±0.82	1.65 ±0.48	0.01 ±0.01
V-Dien Bien	*A*.*c*	Vietnam	1	5	73	12.83 ±2.10	4.90 ±0.57	0.18 ±0.02
V-Son La	*A*.*c*	Vietnam	1	2	5	2.33 ±0.42	2.30 ±0.41	0.16 ±0.09
V-Cat Ba	*A*.*c*	Vietnam	3	4	6	3.83 ±0.60	3.28 ±0.49	0.11 ±0.03

Sample size and population genetic estimators for each sampling location (P for Philippines and V for Vietnam, followed by the closest city to the sampled apiaries) and host species (*A*.*c*: *A*. *cerana*, *A*.*m*: *A*. *mellifera*) on which the mites were collected, together with the clusters provided by the Principal Component Analysis. Number of apiaries *(* ***N***_***A***_); Number of colonies (***N***_***C***_); Number of individual mites (***N***_***I***_); Mean number (± se) of alleles over the six microsatellite markers (**N**_**al**_); allelic richness ± se; mean observed heterozygosity ± se (**H**_**O**_).

### Mitochondrial DNA analysis

A 950bp fragment of the mitochondrial Cytochrome Oxidase I gene (*coxI*) was amplified and sequenced using the *coxI* primer set (10KbCOIF1 and 6,5KbCOIR, [14]) for three mites per host species and location. The resulting sequences were trimmed using the software BIOEDIT [16] and subsequently aligned with the software MEGA V. 6.0 [17]. These fragments were compared with the NCBI database using the NCBI-BLAST tool [18] to infer which of the previously described *V*. *destructor* haplotypes best matched our samples.

The amount of divergence between all distinct haplotypes generated in this study was calculated using the software MEGA V 6.0 [17]. In parallel, a maximum likelihood tree was built using the same software. Finally, a median-joining network was constructed using the software NETWORK v. 4.6.1.2 [19].

### Microsatellite DNA analyses

All *Varroa* were genotyped at six polymorphic microsatellite DNA loci (VD112, VD125, VD152 from [20], and VJ275, VJ292 and VJ295, from [13]) using the Fragment Profiler software V. 1.2 of the MEGABACE DNA Analysis System (GE Healthcare Life Science, Buckinghamshire, England). The number of alleles (NA), allelic richness (R) and the observed heterozygosity (H_O_) were estimated for each sampling location and host species using the software Fstat V. 2.9.3 [21]. Hardy-Weinberg equilibrium tests were performed within samples for each marker using the former software.

A Principal Component Analysis was conducted on the overall microsatellite data using the R package Adegenet [22] to identify the main genetic clusters among the different mite samples based on the individual mites’ genotypes. The fixation indexes (F_ST_) between and within the main clusters provided by the PCA analysis were estimated using Fstat V. 2.9.3 [21]. In addition, Jost’s population differentiation index (*D*, [23]) was estimated using the software SMOGD [24] between all locations and honeybees host species. Finally, AMOVAs were performed using the microsatellite data to identify the relevant level of *Varroa* genetic structuring within the PCA clusters (between locations, between colonies within locations and within colonies) using the software Arlequin V. 3.5.1.3 [25].

## Results

### Mitochondrial DNA analysis

All sequences generated in this study were registered in the NCBI database under accession numbers KR528378 to KR528387 (S1 Table).

#### Origins of the mites from Vietnam

**Fig 1:**
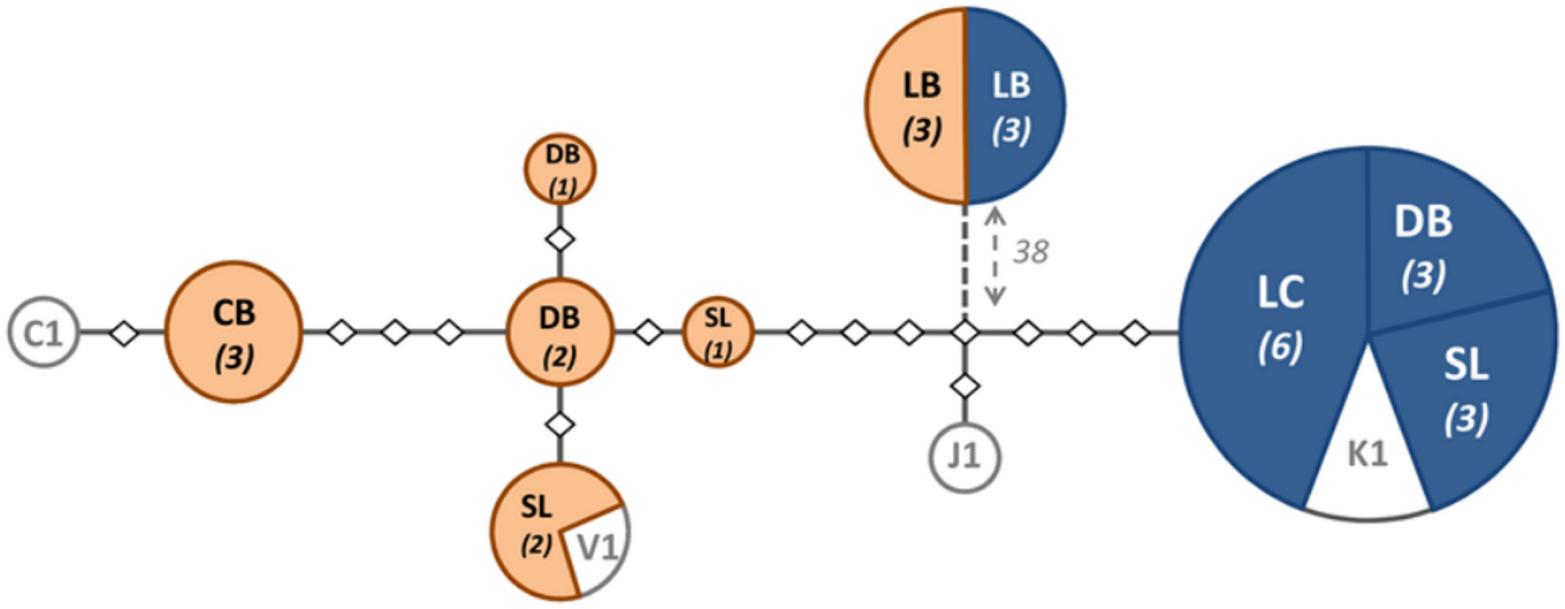
Varroa *coxI* haplotype divergence in the Philippines and Vietnam. Network representing the amount of substitutions between the different *coxI* sequences obtained from different *Varroa* mites sampled in colonies of *A*. *mellifera* (in blue with white text) and *A*. *cerana* (in orange with black text). The mites were sampled in Vietnam in the surrounding of Dien Bien (**DB**), Son La (**SL**) and Cat Ba (**CB**) and in the Philippines in the city of Los Banos (**LB**) and Lipa City (**LC**). The sample size for each haplotype is written in italic between brackets below the sampling location. The grey components represent additional accessions generated by Navajas *et al*. (2010): AmK1-1 haplotype (**K1**, accession GQ379056), AcV1-1 (**V1**, accession GQ379061), AcC1-1 (**C1**, accession GQ379065) and AmJ1-6 together with AcJ1-4 (**J1**, accessions GQ379074.1 and GQ379072.1, respectively). Each diamond represents a substitution. The number indicated close to the dotted line represents the number of substitutions not represented on the figure.

The mtDNA sequences of the Vietnamese samples clearly segregated according to host species. Both haplotypes were however highly similar (Fig 1 and S1 Fig).

The sequences of the Vietnamese mites sampled on *A*. *mellifera* colonies were all identical to the previously described Korean AmK1-1 haplotype (accession GQ379056; [14]). The mites from *A*. *cerana* colonies showed more variability both within and between locations (Fig 1, Table 2). The previously described haplotype AcV1-1 (accession GQ379061; [14]) matched the haplotype of mites sampled on *A*. *cerana* in Dien Bien and Son La. Our samples from Cat Ba were close to the AcC1-1 haplotype from Guangdong province, in Southern China (accession GQ379065; [14]). The haplotypes in Vietnam were also similar to the Japanese haplotype (accession GQ379074.1; [14]) with only five substitutions between the haplotypes of mites sampled at Son La. This was in the same range as the variance found within the Vietnamese samples where the haplotypes of the mites sampled at Cat Ba differed by five substitutions to the Son La haplotype. The distances within the Vietnamese haplotypes were not significantly larger than those separating the Korean and the Japanese haplotype (*t* test, p > 0.05) but significantly larger than the haplotypes sampled on the Phillipines (*t* test, p < 0.001) (Table 2).

**Table 2:** Divergence between *Varroa* Haplotypes.

Country	Host	Haplotypes	Vm_K1	Vc_V1	Vc_C1	Vm-Vc_P
**Vietnam**	**A. m**	**Vm_K1**				
	**A. c**	**Vc_V1**	0.006(**± 0.003)**			
	**A. c**	**Vc_C1**	0.008(**± 0.004)**	0.003(**± 0.002)**		
**Philippines**	**A. m & A. c**	**Vm-Vc_P**	0.035(**± 0.013)**	0.036(**± 0.013)**	0.035(**± 0.013)**	
**Thailand**	**A. c**	**Vj**	0.033(**± 0.012)**	0.035(**± 0.012)**	0.033(**± 0.012)**	0.032(**± 0.012)**

Level of divergence between the different *Varroa coxI* haplotypes found in this study and the main haplotypes from Navajas *et al*. (2010). This table includes the Korean haplotype (**Vm K1**, including the mites sampled in *A*. *mellifera* colonies in Vietnam and Lipa city, and the accession GQ379056), the mainland Vietnamese haplotype (**Vc_V1**, which comprises the mites sampled in *A*. *cerana* colonies in Dien Bien and Son La and the accession GQ379061), the Chinese haplotypes (**Vc_C1**, including the mites sampled in *A*. *cerana* colonies in Ca Ba and accession GQ379065), the native Philippine haplotype (**Vm-Vc_P**, including the mites sampled in both hosts’ colonies in Los Banos) and the *V*. *jacobsoni* sequence (**Vj**, accessions GQ387679.1 and GQ387678.1). The arithmetic mean of all pairwise distances between groups (number of substitutions divided by total sequence length) and their respective standard deviation after 1000 bootstrap between brackets.

#### Origins of the mites from the Philippines

The *coxI* sequences we obtained from the mites sampled in *A*. *cerana* and *A*. *mellifera* pupae cells in Los Banos were all identical to the Luzon 1 sp. (accession AF106894.1, [7]). In that location, the mites we sampled all shared the identical native mite haplotype irrespective of host species, unlike in Lipa city where all mites sampled in *A*. *mellifera* colonies were of the ubiquitous Korean AmK1-1 haplotype (accession number GQ379105.1; [14]).

#### Differences between the mites from Vietnam and Los Banos

The divergence levels between the mites we sampled in Los Banos and in Vietnam were high, with the sequences differing at the average by 3.50% ± 1.30 SD and 3.60% ± 1.30 SD from the *A*. *mellifera* and *A*. *cerana* mites from Vietnam, respectively (Table 2).

### Microsatellite markers Analysis

The microsatellite markers used in this study were highly polymorphic with an average of 18.83 ± 3.33 alleles (Table 3). None of the six markers were in Hardy-Weinberg equilibrium, due to a lack of heterozygotes which is expected as a result from obligate brother-sister mating and inbreeding in the *Varroa* life cycle.

**Table 3:** Overall information on the microsatellite loci used.

Locus	NA	R
**VD112**	16	3.07
**VD126**	10	2.27
**VD152**	12	3.60
**VJ275**	31	6.10
**VJ292**	26	3.28
**VJ295**	18	2.97

For each of the six microsatellite markers used, the name of the locus (**Locus**), the overall number of Alleles (**N**_**A**_) and the overall Allelic Richness (**R**) are represented.

#### Principal Component analysis

**Fig 2:**
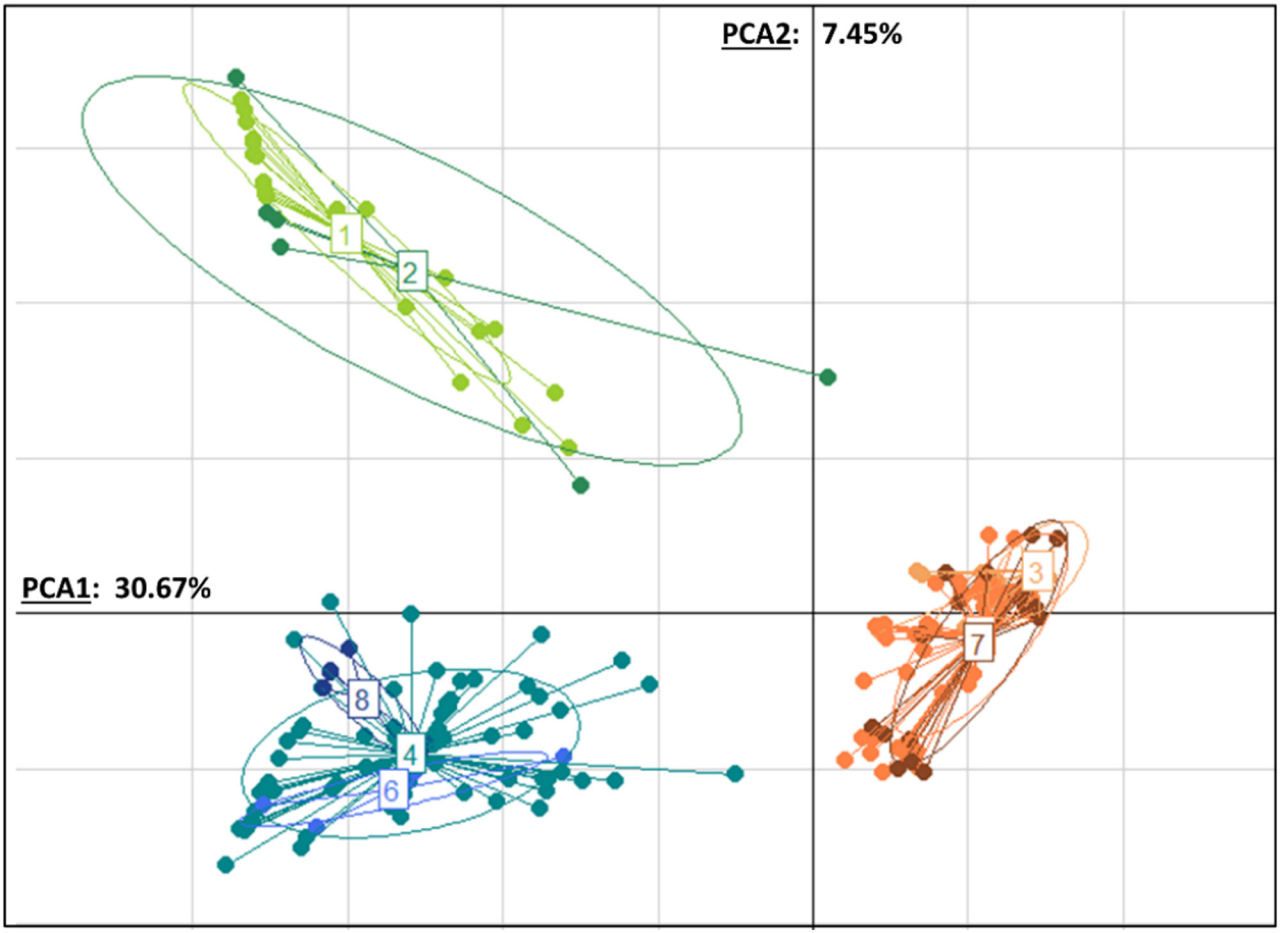
Principal Component Analysis. Genetic clustering of the *Varroa* mites based on their genotype at six microsatellite markers. The *Varroa* mites were sampled in *A*. *cerana* (**Vc**) and *A*. *mellifera* (**Vm**) colonies in the Philippines and Vietnam. The genotypes of the mites sampled in Los Banos (**LB**) are shown in green (groups 1 and 2). The mites from Lipa city (**LC**, group 3) and sampled in *A*. *mellifera* from Dien Bien (**DB**, group 5) and Son La (**SL**, group 7) are represented in orange. The mites from *A*. *cerana* colonies located in Vietnam from Dien Bien (**DB**, group 4), Son La (**SL**, group 6) and Cat Ba (**CB**, group 8) are represented in blue. Each dot represents a distinct individual, and each inertia ellipsoid shows the population’s prediction ellipses for each group.

Based on the microsatellite data of all mites, the two first components obtained with the PCA explained together 38.32% of the genetic variation in our samples (first component: 30.67% and second component: 7.45%, Fig 2). When the individual mite genotypes were plotted on these two main axes, three distinct clusters could be observed, matching the three different haplotype described with the mitochondrial DNA analysis. The first one consisted of the mites sampled in *A*. *cerana* colonies in the three populations in Vietnam (“Vietnamese cluster”). A second cluster included the *Varroa* collected in *A*. *mellifera* in Vietnam and Lipa city (“Korean cluster”). Finally, the third cluster comprised the parasites from the colonies of *A*. *mellifera* and *A*. *cerana* from Los Banos (“Philippine cluster”).

#### Comparison of the Genetic Diversity between the different Varroa types

The mites belonging to the Vietnamese type had a significantly higher allelic richness (14.13 ± 2.04) compared to the *Varroa* mites of the Korean cluster (2.84 ± 1.39) and the Philippine cluster (R = 3.63 ± 0.91, *t* test: *p* = 0.001) (Table 1). The overall heterozygosity in the mites was low. However, it was almost an order of magnitude higher in the *Varroa* sampled in *A*. *cerana* colonies in Vietnam (average H_O_ = 0.17 ± 0.02 SD) and in Los Banos (H_O_ = 0.16 ± 0.06) than in the mites from the Korean cluster (average H_O_ = 0.02 ± 0.02, *t* test, *p* < 0.05).

#### Genetic structuring of the Varroa haplotypes

The genetic differentiation within the clusters was low and non-significant for the mites sampled in *A*. *cerana* and *A*. *mellifera* colonies in Los Banos (F_ST_ = 0.052, *p* > 0.05) (Table 4). However, medium and significant F_ST_ values were obtained when comparing the different sampling locations where the Korean cluster was found (F_ST_ = 0.172, *p* < 0.05) and the different sampling locations where the Vietnamese cluster was found (F_ST_ = 0.205, *p* < 0.05). Much higher and highly significant F_ST_ values were found when comparing the three clusters (Table 4).

**Table 4:** Pairwise F_ST_.

Clusters	Philippines	Korea	Vietnam
**Philippines**	0.052		
**Korea**	0.749***	0.172***	
**Vietnam**	0.315**	0.520***	0.205**

Pairwise comparison of the genetic differentiation using the fixation index (F_ST_) between the three main genetic clusters found in this study: the mites from the two honeybees species sampled in Los Banos (**Philippines**), the mites sampled in *A*. *mellifera* colonies in Vietnam and Lipa city (**Korea**) and the mites sampled in *A*. *cerana* colonies in Vietnam (**Vietnam**).

***: *p*<0.001,

**: *p*<0.01.

The AMOVA revealed that the geographic location had a highly significant importance for the mites of theVietnamese cluster (15.03%, *p* < 0.001) and in the Korean cluster (15.12%, *p* < 0.01) (Table 5). Notably, the genetic distance in the Korean cluster was lower within the two Vietnamese populations (D = 0.008) than between the mites from Lipa city and the two Vietnamese populations (D = 0.023) (S2 Table). The amount of genetic diversity varied significantly within location for both, the Vietnamese and the Korean clusters (16.72% and 15.85%, respectively, *p* < 0.001). Finally, the largest source of variation in these two groups resulted from the differences among mites within colonies (68.24% for the Vietnamese and 69.02% for the Korean clusters, *p* < 0.001). For the mites sampled in Los Banos, only this latter level was significant (80.47%, *p* < 0.05), but not the differences among hosts or among colonies within host.

**Table 5:** Results of the Analyses of Molecular Variance.

Cluster	Source of variation	d.f.	Sum of sq.	Var. comp.	% Variation	*p*
**Vietnamese**	Among Locations	2	26.04	0.43	15.03	***
	Within location	14	89.09	0.47	16.72	***
	Within colonies	151	269.98	1.94	68.24	***
	**Total**	167	385.12	2.84		
**Korean**	Among Locations	1	3.06	0.01	15.12	**
	Within location	13	39.89	0.13	15.85	***
	Within colonies	317	149.86	0.52	69.02	***
	**Total**	513	192.81	0.65		
**Philippines**	Among hosts	1	3.65	0.05	3.46	N.S.
	Among colonies within host	4	12.57	0.24	16.07	N.S.
	Within colonies	56	56.30	1.21	80.47	*
	**Total**	61	72.52	1.50		

Table representing the results of the different AMOVA performed with the software Arlequin for the three clusters considered: **Vietnamese**: mites sampled in *A*. *cerana* colonies in Vietnam, **Korean**: mites sampled in *A*. *mellifera* colonies in Vietnam and Lipa city and **Philippines**: mites sampled in Los Banos in colonies of both host species.

***: p<0.001,

**: p <0.01,

*: p<0.05,

N.S.: non-significant.

#### Hybrid detection

We found no evidence of direct hybridization between mites of the two host species. Only eight *Varroa* mites in the whole dataset carried alleles that were also found in mites sampled in the alternative host species. These individuals were exclusively found in *A*. *cerana* colonies in Vietnam and carried one or two alleles that were otherwise specific to the Korean haplotype in *A*. *mellifera* colonies. However, none of these mites were direct hybrids, as all other microsatellite loci had private alleles of the Vietnamese haplotype. Moreover, these few shared alleles were found in the homozygotic state, suggesting that they were independent homoplasic alleles of the same length as in the *A*. *mellifera* sampled mites but not a result of hybridisation.

## Discussion

In this study, we found that the *Varroa* mite shows different patterns of host specificity between *A*. *cerana* and *A*. *mellifera* in the Philippines and in Vietnam. Whereas the native Philippine mite was found in colonies of both host species in Los Banos, we found strong host specificity and complete reproductive isolation between the *Varroa* types parasitizing *A*. *cerana* and *A*. *mellifera* colonies in Vietnam. The Korean haplotype was only found in *A*. *mellifera* colonies but never found in any *A*. *cerana* colony we sampled in Vietnam and the Philippines.

### Origins and diversity of the Varroa mites from Vietnam

Our findings support the suggestion of Fuchs *et al*. [26], who reported on an almost complete host specificity of the two *Varroa* lineages in Northern Vietnam, suggesting sympatry of two host specific *Varroa* types that do not hybridize. Despite only a minute divergence of the Vietnamese *coxI* haplotype from the Japanese and also the Korean *V*. *destructor* haplotype, our nuclear DNA analyses suggest a complete genetic isolation of the mites from the different host species. Not only did we not detect any indication of hybridization, we also failed to sample mites typical for the one host species in colonies of the other host species in Vietnam. Hence the Korean haplotype found in *A*. *mellifera*, which may have switched hosts only about 60 years ago [4], is not able to infect different populations of its original host species established in Northern Vietnam.

Our results show that the arm race between *Varroa* and its hosts has led to the evolution of very specialized mites. Although the underlying mechanisms of this coevolution are not well understood, previous work suggests that the mite is able to mimicry the cuticular hydrocarbons of its host [27–28] to avoid the hygienic behaviour of the honeybees [29]. Even though this trait appears to be plastic [30], the Korean haplotype is apparently not able to overcome the defenses of the populations of *A*. *cerana* found in Vietnam.

The level of genetic diversity and genetic structuring among the three sampling locations in Vietnam were higher in the mites sampled in *A*. *cerana* colonies than between the two locations where we sampled in *A*. *mellifera* colonies. This matches reports of the global spread of very few, genetically almost identical *V*. *destructor* lineages in *A*. *mellifera* [13].

### Origins and diversity of the Varroa mites from the Philippines

The archipelago of the Philippines accommodates distinct and diverse *A*. *cerana* host populations that show haplotype variation at the subspecies level compared to mainland Asian populations [31–32]. This clearly sets the stage for independent coevolution between mites and hosts and may explain the large genetic differences between mainland Asia and the Philippine mites previously described by Anderson and Trueman [7]. In that study, *Varroa* from three provinces of the Philippines were analyzed: *A*. *cerana* colonies were sampled in two provinces on the island of Luzon: Batangas (which is the adjacent province located South of our sampling location) and San Fernando (a province located about 100 km to the North of our sampling location), and a third one in Mindanao, a different island. Each of these three sampling locations hosted a distinct mite haplotype in *A*. *cerana* colonies, grouping apart from the rest of the *V*. *destructor* sequences coming from *A*. *cerana* mites sampled in other Asian countries. However, the mites sampled from *A*. *mellifera* colonies in the Philippines all shared the Korean haplotype suggesting a separation of native and introduced mite populations as we observed in Vietnam in this study. We also found that the sequences of the mites we sampled in *A*. *cerana* in the Philippines differed significantly from the rest of the haplotypes previously described [14]. However, contrary to Anderson and Trueman [7], we also found the Luzon 1 haplotype in the Philippine *A*. *mellifera* colonies in Los Banos.

In addition to the lack of mitochondrial DNA variability in Los Banos, we also failed to detect any level of subpopulation structuring in this *Varroa* population. In fact, the microsatellite markers we analyzed suggested that the mites readily infect both host species. The presence of the native Philippine type in *A*. *mellifera* shows that more types than previously thought may be able to infect both *Apis* species.

### A clearer picture on the Varroa genetic and functional diversity

By coupling both mitochondrial and nuclear DNA approaches, we were able to infer the origin of the mites, but also to detect more functional mechanism such as the hybridization potential of different *Varroa* types in their native and non-native range. The substantial differences between the genetic diversity and infestation abilities of the mites sampled in Los Banos and in Vietnam may have far reaching consequences for our understanding of the host parasite biology of honeybees and *Varroa*.

The mites we sampled in Los Banos show all genetic prerequisites to qualify as a novel *Varroa* species. Although Anderson and Trueman [7] did not find morphological distinctiveness (based on body size) to completely separate this “Luzon haplotype 1” to the other *Varroa* species, we found further evidence that the mites from Los Banos differ markedly from the four other previously described *Varroa* species. Both mitochondrial DNA sequence divergence and the ability to parasitize both *A*. *cerana* and *A*. *mellifera* render this *Varroa* type a potential novel species. In addition, however, also the *Varroa* types we found in Vietnam appear to segregate as if they were distinct species. Although the mitochondrial haplotypes were rather similar to the Japanese haplotype of *V*. *destructor*, the microsatellite DNA markers showed a complete separation of these two mite groups even if kept on the same apiary. Thus, despite the fact that the number of substitutions is well below the 2% *coxI* divergence level typically considered as separating two species [33], there is no indication of any hybridization of nuclear markers. Given the Biological Species Concept of the reproductive isolation between the two sympatric groups [34–35], the mites with the Korean and the Vietnamese haplotypes could also be considered as two distinct species.

Solignac *et al*. [13] estimated the divergence time between the Korean and the Japanese *Varroa* type between 5 000 and 15000 years ago. Assuming a constant mutation rate and population size, we can estimate the divergence time among the various haplotypes found in our study. Four substitutions in the cox I sequence separate the Japanese from the Korean haplotype [14] resulting in an estimate 1250 and 3750 years of divergence per substitution. Similarly, the time of divergence between the two types found in Vietnam (which differ in by between five and eight substitutions) from the Japanese type would be between 10 000 and 30 000 years. Considering the short generation time of the *Varroa* mite [3], this is a rather long speciation period: This result could explain why the ubiquitous Korean haplotype can no longer parasitize *A*. *cerana* in Vietnam in spite of ongoing spill overs and spill backs in Korea and in Japan [14]. Following this reasoning, 50000 to 150000 years would separate the mites from the Philippines and the two Vietnamese haplotypes. This falls within the Pleistocene, during which *A*. *cerana* may have arrived in archipelago of the Philippines [31–32]. The ancestor of the *Varroa* mite found in the Philippines nowadays may have evolved in allopatry from the mainland populations since then.

## Conclusions

We here provide an example of how host-parasite coevolution can rapidly lead to speciation within a short time span. The initial extreme selection on the Korean mites after the host switch has allowed for only a very limited number of individuals to reproduce in the novel host species less than a century ago. Subsequently, the constant brother-sister mating of *Varroa* has led to almost clonal population-specific mite types, which have differentiated considerably with time as they were taken away from their native region into allopatry. These extreme characteristics of the mite set the stage for the potential alloxenic speciation [36] of highly specific and most virulent types.

The consequences of keeping the introduced Western honeybee and the native Asian species is a most unfortunate example of transhumance having devastating consequences by promoting the global spread of parasites and associated viruses [8, 37–39]. Since apiculture has facilitated the global transmission of *Varroa*, selection will inevitably favor the most virulent types in *A*. *mellifera* as seen for the global spread of the Korean haplotype. Yet, given the tremendous increase of *A*. *mellifera* beekeeping in Asia and the wide diversity of *Varroa* in the native *A*. *cerana* populations, it seems possible that more mite types might switch to the western honeybee. At the same time, the spill back of virulent *Varroa* strains from *A*. *mellifera* to *A*. *cerana* may also become a risk potential for apiculture for these two economically and ecologically crucial species. Although some Varroa types are apparently strongly specific (Vietnam), others are more generalist (Philippines). If those generalist mites would spread to mainland Asia, it is likely that they would also invade both *A*. *mellifera*, but also *A*. *cerana* colonies.

## Supporting Information

S1 Fig Maximum likelihood Tree. Phylogenetic tree representing the sequences generated in this study and the sequences generated with the same primers by Navajas *et al*. (2010). The tree is based on a partial deletion model, with nodes representing values for 1000 bootstraps. **Viet**: samples from Vietnam (this study); **SL**: Son La, **DB**: Dien Bien, **CB**: Cat Ba; **Phil**: samples from the Philippines (this study); **Nav**: from Navajas *et al*. (2010).
(TIF)

S1 Table Information on the Accessions (DOCX)

S2 Table Results of the Population Differentiation between hosts and sampling locations (Jost’s D). (DOCX)
