# Supplementary figures and images for: Host Specificity in the Honeybee Parasitic Mite, Varroa spp. in Apis mellifera and Apis cerana
Source: PLoS One. 2015 Aug 6;10(8):e0135103. doi: 10.1371/journal.pone.0135103 (PMC4527838; doi:10.1371/journal.pone.0135103)

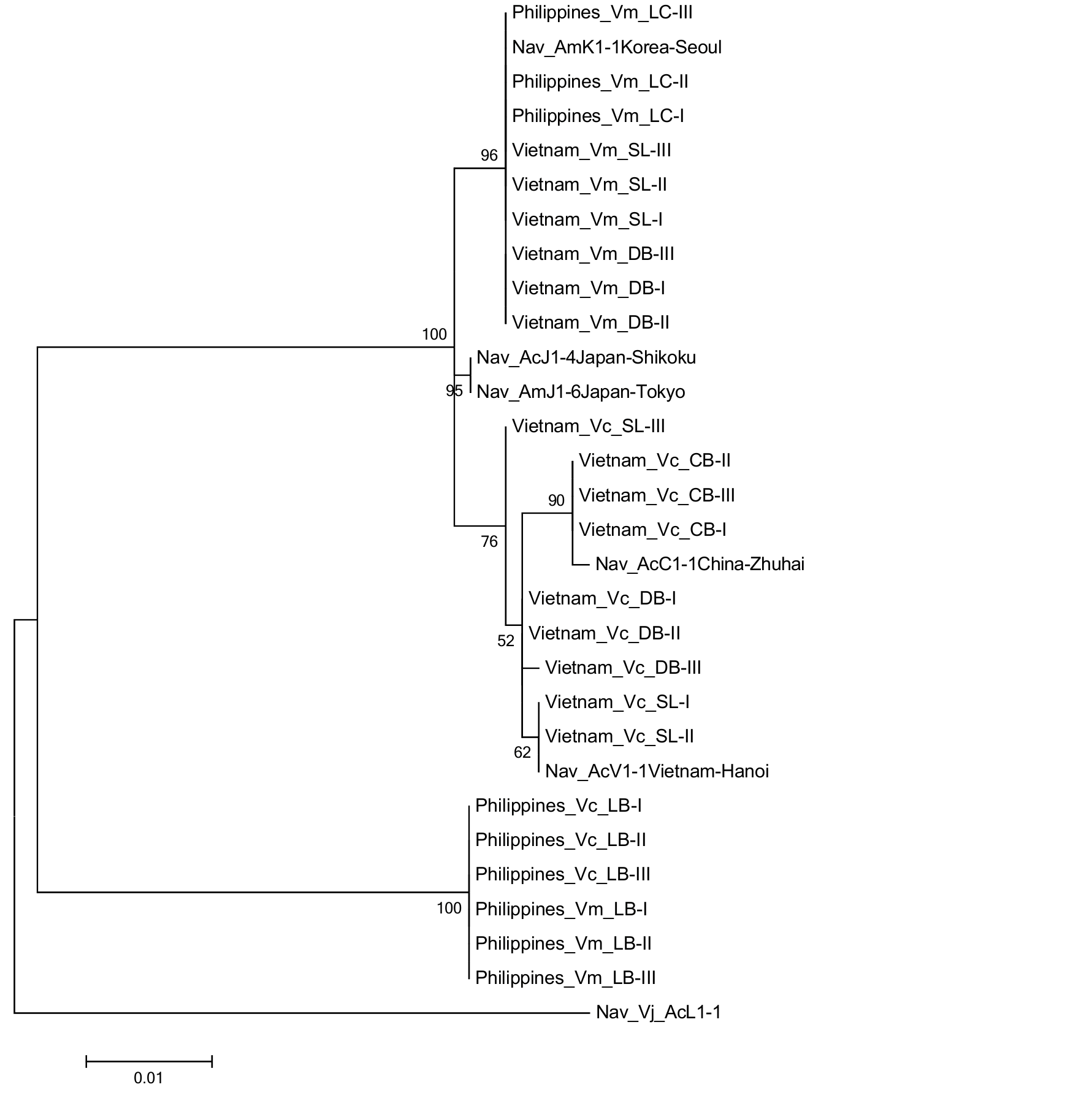

Supplement: S1 Fig — Phylogenetic tree representing the sequences generated in this study and the sequences generated with the same primers by Navajas et al. (2010). The tree is based on a partial deletion model, with nodes representing values for 1000 bootstraps. Viet: samples from Vietnam (this study); SL: Son La, DB: Dien Bien, CB: Cat Ba; Phil: samples from the Philippines (this study); Nav: from Navajas et al. (2010). (TIF) [file pone.0135103.s001.tif]
