# Supplementary material for: Host Specificity in the Honeybee Parasitic Mite, Varroa spp. in Apis mellifera and Apis cerana
Source: PLoS One. 2015 Aug 6;10(8):e0135103. doi: 10.1371/journal.pone.0135103 (PMC4527838; doi:10.1371/journal.pone.0135103)
Supplement: S1 Table — (DOCX) [file pone.0135103.s002.docx]

S1 Table. Information on the Accessions

| Country | Location | Host | Date | Lat_Lon | Accession N° |
| --- | --- | --- | --- | --- | --- |
| Viet Nam | Cat Ba | A. cerana | 11-11-13 | 20.43 N 107.03 E | KR528378 |
|  | Dien Bien | A. cerana | 05-11-13 | 21.23 N 103.01 E | KR528379 |
|  | Dien Bien | A. cerana | 05-11-13 | 21.23 N 103.01 E | KR528380 |
|  | Dien Bien | A. mellifera | 05-11-13 | 21.23 N 103.01 E | KR528381 |
|  | Son La | A. cerana | 07-11-13 | 21.19 N 103.54 E | KR528382 |
|  | Son La | A. cerana | 07-11-13 | 21.19 N 103.54 E | KR528383 |
|  | Son La | A. mellifera | 07-11-13 | 21.19 N 103.54 E | KR528384 |
| Philippines | Lipa city | A. mellifera | 02-02-15 | 13.56 N 121.09 E | KR528385 |
|  | Los Banos | A. cerana | 04-04-13 | 14.09 N 121.14 E | KR528386 |
|  | Los Banos | A. mellifera | 04-04-13 | 14.09 N 121.14 E | KR528387 |

Information on the accessions generated in this study: country of sampling (Country), closest city to the sampled apiaries (location), honeybee species where the mites were collected (Host), date of collection (Date), coordinates (Lat Lon) and NCBI accession number (Accession N°).
