# Supplementary material for: Host Specificity in the Honeybee Parasitic Mite, Varroa spp. in Apis mellifera and Apis cerana
Source: PLoS One. 2015 Aug 6;10(8):e0135103. doi: 10.1371/journal.pone.0135103 (PMC4527838; doi:10.1371/journal.pone.0135103)
Supplement: S2 Table — (DOCX) [file pone.0135103.s003.docx]

S2 Table. Results of the Population Differentiation between hosts and sampling locations (Jost’s D).

|  | **LB_Vc** | **LB_Vm** | **LC_Vm** | **DB_Vc** | **DB_Vm** | **SL_Vc** | **SL_Vm** |
| --- | --- | --- | --- | --- | --- | --- | --- |
| **LB_Vm** | 0.066 |  |  |  |  |  |  |
| **LC_Vm** | 1.000 | 0.826 |  |  |  |  |  |
| **DB_Vc** | 0.992 | 0.956 | 0.747 |  |  |  |  |
| **DB_Vm** | 1.000 | 0.827 | 0.023 | 0.746 |  |  |  |
| **SL_Vc** | 1.000 | 1.000 | 0.722 | 0.225 | 0.722 |  |  |
| **SL_Vm** | 1.000 | 0.830 | 0.023 | 0.744 | 0.008 | 0.721 |  |
| **CB_Vc** | 1.000 | 1.000 | 0.859 | 0.541 | 0.859 | 0.528 | 0.858 |

Table representing the pairwise Jost’s D estimate calculated between the *Varroa* mites coming from the two honeybee hosts species (*A. cerana*: Vc and *A. mellifera* Vm) between two locations in the Philippines (LB: Los Banos, LC: Lipa City) and three locations in Vietnam (DB: Dien Bien, SL: Son La and CB: Cat Ba).
